# DDR1 promotes E-cadherin stability via inhibition of integrin-β1-Src activation-mediated E-cadherin endocytosis

**DOI:** 10.1038/srep36336

**Published:** 2016-11-08

**Authors:** Hong-Ru Chen, Yi-Chun Yeh, Ching-Yi Liu, Yu-Ting Wu, Fang-Yu Lo, Ming-Jer Tang, Yang-Kao Wang

**Affiliations:** 1 Department of Physiology National Cheng Kung University, Tainan, Taiwan; 2 Institute of Basic Medical Sciences, National Cheng Kung University, Tainan, Taiwan; 3 Department of Cell Biology and Anatomy, College of Medicine, National Cheng Kung University, Tainan, Taiwan

## Abstract

Discoidin domain receptor 1 (DDR1), a receptor tyrosine kinase of collagen, is primarily expressed in epithelial cells. Activation of DDR1 stabilises E-cadherin located on the cell membrane; however, the detailed mechanism of DDR1-stabilised E-cadherin remains unclear. We performed DDR1 knockdown (Sh-DDR1) on Mardin-Darby canine kidney cells to investigate the mechanism of DDR1-stabilised E-cadherin. Sh-DDR1 decreased junctional localisation, increased endocytosis of E-cadherin, and increased physical interactions between E-cadherin and clathrin. Treatment of the dynamin inhibitor Dyngo 4a suppressed Sh-DDR1-induced E-cadherin endocytosis. In addition, the phosphorylation level of Src tyrosine 418 was increased in Sh-DDR1 cell junctions, and inhibition of Src activity decreased Sh-DDR1-induced E-cadherin endocytosis. To characterise the molecular mechanisms, blocking integrin β1 decreased Src activity and E-cadherin junctional localisation in Sh-DDR1 cells. Photoconversion results showed that inhibition of Src activity rescued E-cadherin membrane stability and that inhibition of integrin β1-Src signalling decreased stress fibres and rescued E-cadherin membrane stability in Sh-DDR1 cells. Taken together, DDR1 stabilised membrane localisation of E-cadherin by inhibiting the integrin β1-Src-mediated clathrin-dependent endocytosis pathway.

Adherens junctions are cell-cell adhesion complexes that produce strong mechanical attachments between adjacent cells and that cause cells to function as a unit. Zonula adherens is a type of adherens junction that exists in epithelial cells; it completely encircles the apex of the epithelial cells, linking them into a sheet and separating the apical and basolateral membranes of each highly polarised cell^1^,^2^,^3^. E-cadherin is the core component of zonula adherens and plays a crucial role in maintaining epithelial differentiation and cell polarity^4^. Therefore, loss of E-cadherin has been identified as the hallmark of epithelial-mesenchymal transition (EMT), which is a critical process involved in cancer metastasis^5^,^6^,^7^,^8^. In addition, EMT is a key mechanism for organ fibrosis^6^,^9^,^10^,^11^, and wound healing and the turnover of rapidly growing tissues in adult cells are also involved in EMT^12^. Therefore, regulation of E-cadherin-based junctional stability controls cell behaviour.

The silencing of E-cadherin gene expression typically results in permanent loss of zonula adhesion. The genetic and epigenetic alterations of an E-cadherin locus highly correlate with malignancy in various types of human cancers^13^,^14^,^15^,^16^,^17^. Besides controlling E-cadherin gene expression, the stability and endocytosis of E-cadherin play a critical role in controlling its protein levels at adherens junctions. Previous studies have shown that the association between receptor tyrosine kinases (RTKs) and the E-cadherin-catenin complex causes the endocytosis of E-cadherin with RTKs when ligand binding is performed^18^,^19^. The phosphorylation of E-cadherin at Ser684, Ser686, and Ser692 by glycogen synthase kinase 3β and casein kinase 2 increases its binding affinity with β-catenin^20^, and phosphorylation of β-catenin at Tyr489, Tyr654, or Tyr142 disrupts binding to cadherin and α-catenin, thereby reducing junctional stability^21^,^22^,^23^. In addition, phosphorylation of E-cadherin at Tyr755 and Tyr756 disrupts the binding of p120 to E-cadherin, thus causing the ubiquitination and degradation of E-cadherin^24^,^25^,^26^. Cis-homodimeric E-cadherin is more stable than trans-homodimeric E-cadherin because cis-homodimeric E-cadherin forms lateral clustering^27^ that is supported and maintained by actin patches^28^. Because of its diversity and complexity, the molecular mechanisms regulating the stability of E-cadherin are not fully understood.

Previous studies have demonstrated that an increase in a discoidin domain receptor 1 (DDR1) signal promotes epithelial differentiation and cell polarity^29^. DDR1 belongs to a specific protein family named the discoidin domain receptor (DDR), which was discovered using homology cloning in the search for new RTKs. The name DDR is used because this protein contains discoidin homology domain that was first described in the slime mould *Dictyostelium discoideum* as Discoidin I^30^, and DDR1 was ultimately identified as a type of collagen receptor^31^. Two types of members are present in the DDR family: DDR1 is primarily expressed in epithelial cells and DDR2 is primarily expressed in stromal cells^32^. Overexpression of DDR1 reduces collagen-induced cell proliferation, extension, and migration, whereas overexpression of dominant negative DDR1 produces an increase in these processes^33^,^34^,^35^. These studies have indicated that DDR1 plays a crucial role in epithelial cell differentiation.

In addition to the phosphorylation of E-cadherin in the regulation of adherens junctions, previous studies have demonstrated that the expression of DDR1 increases the membrane localisation of E-cadherin, which results in the resistance of E-cadherin to collagen-induced endocytosis^36^. Moreover, the expression of DDR1 reduces the turnover rate of E-cadherin^29^. By using E-cadherin conjugated with mEos fluorescence protein, the expression of DDR1 decreases the lateral diffusion rate and increases membrane stability of E-cadherin^29^. However, the signal transduction pathway that DDR1 uses to inhibit E-cadherin endocytosis is unclear.

The purpose of this study was to identify the signalling transduction pathway of DDR1-regulated E-cadherin membrane stabilisation. According to previous studies, one of the endocytosis pathways involved in E-cadherin endocytosis is mediated by the activation of Src, which triggers the phosphorylation of E-cadherin on the p120-catenin binding site, as well as E-cadherin endocytosis through a clathrin-mediated pathway^24^,^25^. Src kinase activity and its SH2/SH3 domains are required to impair E-cadherin localisation through MEK/ERK, ROCK, and MLCK pathways^37^. Previous studies have also demonstrated a reciprocal regulation between E-cadherin and integrins^38^. Furthermore, inhibition of Src kinase activity in cancerous colon blocks the phosphorylation of β-catenin tyrosine-654 and prevents the dissociation of β-catenin and E-cadherin^39^. As a result, the present study proposed that DDR1 inhibits the endocytosis of E-cadherin through the regulation of integrin-Src activity. The results may provide a comprehensive mechanism to explain how DDR1 regulates E-cadherin stability and help to clarify the functions of DDR1 during the development of cancerous cells.

## Results

### Knockdown of DDR1 increases E-cadherin endocytosis but does not affect protein abundance

**Figure 1:**
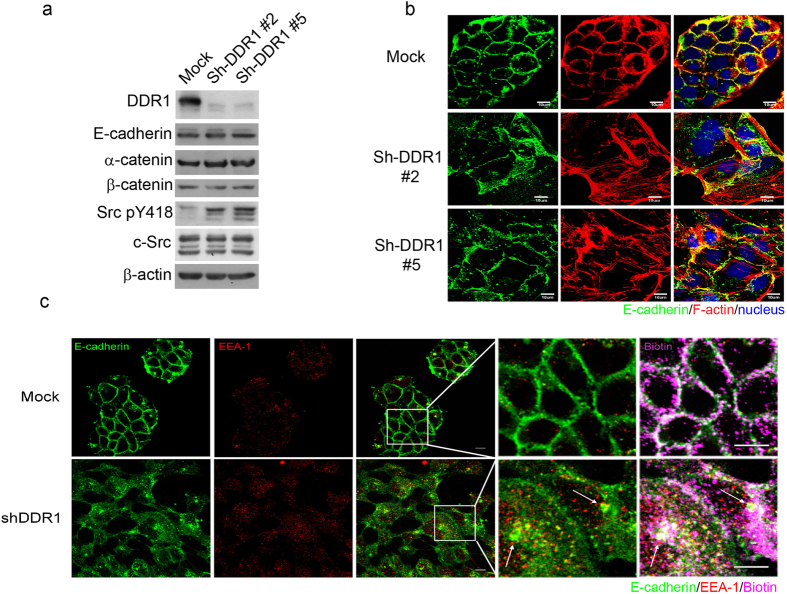
Knockdown of DDR1 decreases junctional localisation and increases endocytosis of E-cadherin. (**a**) Control (Mock) and DDR1 knockdown (Sh-DDR1) MDCK 3B5 cells (Sh-DDR1#2, Sh-DDR1#5) were cultured on a culture dish for 24 h. The protein levels of DDR1, E-cadherin, α-catenin, β-catenin, Src pY418, and total Src were assessed using immunoblotting. (**b**) Localisation of E-cadherin (green) and F-actin (red) in Mock and Sh-DDR1 clones, and images were captured using a confocal microscope (Olympus FV-1000). Bar: 10 μm. (**c**) Mock (upper panels) and Sh-DDR1 (lower panels) cells were cultured on chamber slides for 24 h. The total cell membrane proteins were labelled using biotin, and endocytosis occurred at 37 °C for 30 min. Cells were then fixed and stained with E-cadherin (green) and EEA-1 (red). Biotins (pink) were labelled using Avidin-Alexa 594. Images were captured using a confocal microscope (Olympus FV-1000). Bar: 10 μm. Dashed rectangles were enlarged as indicated in the images on the right-hand side. Arrows indicate the colocalisation of E-cadherin, biotin, and EEA-1. Bars in the enlarged image: 5 μm.

To investigate the mechanism of DDR1-regulated E-cadherin endocytosis, we performed small hairpin RNA on MDCK cells, which were stably transfected using a control vector (Mock) or Sh-DDR1. Two Sh-DDR1 stable clones, denoted as #2 and #5, were selected. After culturing these cells on tissue culture dishes for 24 h, the protein abundance of DDR1, E-cadherin, β-catenin, α-catenin, and Src pY418 was analysed using Western blotting. Sh-DDR1 was efficiently used to decrease the protein levels of DDR1, whereas the protein levels of E-cadherin, β-catenin, and α-catenin were not affected. However, levels of Src pY418 were increased in Sh-DDR1 clones (Fig. 1a). We then investigated whether knockdown of DDR1 would change the subcellular localisation of cell adhesion molecules by using immunostaining. In Mock cells, E-cadherin and F-actin colocalised at cell-cell adhesion sites and formed a clear and continuous cell-cell junction. In contrast, cell size became larger and E-cadherin staining was located primarily in the cytosol, forming a discontinuous cell-cell junction in both Sh-DDR1 clones (Fig. 1b). These knock down effects on cell morphology and cell-cell interaction were similar to a previously published study that used a DDR1 dominant-negative clone^35^. The junctional F-actin disappeared and stress fibres formed in both knockdown clones (Fig. 1b). These results suggested that DDR1 regulates junctional stability and localisation of E-cadherin in epithelial cells.

Our early studies have demonstrated that knockdown of DDR1 increases the colocalisation of cytosolic E-cadherin and early endosome antigen 1 (EEA1), an early endosome marker protein, suggesting that the function of DDR1 is to stabilise cell-cell adhesion by inhibiting the endocytosis of E-cadherin^29^. The expression of DDR1 may also result in the increased recycling process of E-cadherin from the cytosol to the membrane. To elucidate whether DDR1 affected E-cadherin endocytosis, we applied an endocytosis assay^40^. Mock and Sh-DDR1 cells were first labelled with EZ-link NHS-SS-biotin on membrane proteins and placed in a temperature of 37 °C for 30 min to induce endocytosis; the cells were then fixed and stained with E-cadherin, biotin, and EEA1. As shown in Fig. 1c, E-cadherin (green colour) primarily localised at the cell-cell adhesion site in Mock cells (upper panels) to form a continuous border around the cells and colocalising with membrane-biotin staining (purple colour). EEA-1 (red colour) localised primarily in the cytosol and only a limited amount of EEA-1 colocalised with E-cadherin (Fig. 1c, upper panels). However, E-cadherin formed a discontinuous border around Sh-DDR1 cells (Fig. 1c, lower left panels) and the majority of E-cadherin colocalised with biotin and EEA-1 (Fig. 1c, lower panels, arrows) in the endocytic vesicles (Fig. 1c, lower right panel, arrows). These results suggested that DDR1 stabilises E-cadherin at the cell-cell adhesion site by inhibiting endocytosis of E-cadherin. Similar results were obtained using immortalised HPDE and M10 (Supplementary Fig. 1a,b), where knockdown DDR1 in these cell lines resulted in a decrease in junctional localisation of E-cadherin.

### Knockdown of DDR1 increases E-cadherin endocytosis through clathrin-dependent pathway

**Figure 2:**
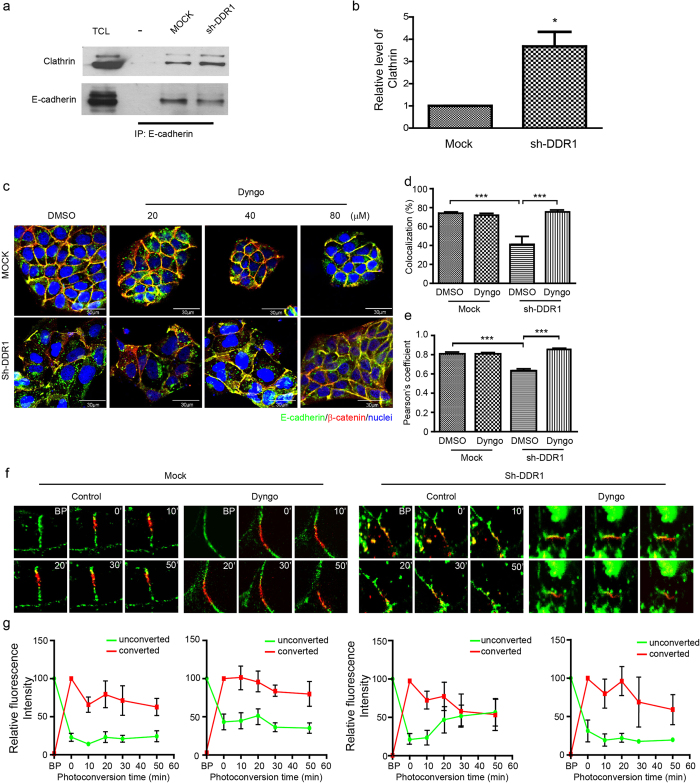
Clathrin-mediated endocytosis is involved in the knockdown of DDR1-induced E-cadherin endocytosis. (**a**) The interaction between E-cadherin and clathrin in Mock or Sh-DDR1 cells was assessed using immunoprecipitation of E-cadherin, followed by immunoblotting with anti-clathrin heavy chain or anti-E-cadherin antibodies. TCL represents total cell lysate; “-” represents immunoprecipitation with the control antibody. (**b**) Quantification results of the ratio of clathrin/E-cadherin intensity. Each bar represents mean ± SE of three independent experiments. *Indicates *P* < 0.05. (**c**) Mock and Sh-DDR1 cells were plated for 24 h and then treated with DMSO or specific dynamin inhibitor Dyngo^®^ 4a (Dyngo) for 4 h at the indicated concentrations. Cells were then fixed and immunostained with E-cadherin (green) and β-catenin (red). Bar: μm. (**d**) The fluorescence intensity profiles of the colocalisation of E-cadherin and β-catenin were assessed using Olympus FV-1000 imaging software. (**e**) The fluorescence intensity profiles of the Pearson’s correlation coefficient of E-cadherin and β-catenin were assessed using Olympus FV-1000 imaging software. (**f**) Mock and Sh-DDR1 cells were transiently transfected with HECD-mEosFP and incubated on chamber slides for 24 h. After receiving DMSO or Dyngo^®^ 4a (Dyngo) treatment for 4 h, Mock and Sh-DDR1 cells were subjected to photoconversion analysis. The HECD-mEos emission wavelengths of 516 nm (green or unconverted) or 581 nm (red or converted) before and after photoconversion by a 405-nm laser was recorded at the indicated times for Mock and Sh-DDR1 cells. The images shown here are the represented images of at least three independent experiments. BP: before photoconversion. (**g**) The quantification results of each photoconversion study are shown underneath each treatment of (**f**).

The above results indicated that knockdown of DDR1 may increase E-cadherin endocytosis. Because endocytosis is divided into clathrin dependent, caveolae dependent (i.e., nonclathrin), and noncaveolae dependent categories^41^, we sought to determine which endocytosis pathway is involved in the knockdown of DDR1-induced E-cadherin endocytosis. Previous studies have demonstrated the possible involvement of a clathrin-mediated endocytosis pathway for the endocytosis of E-cadherin^26^; therefore, we applied immunoprecipitation to examine whether DDR1 affected the interaction between E-cadherin and clathrin. In Mock cells, only limited interaction was shown between E-cadherin and clathrin, and knockdown of DDR1 increased the interaction between E-cadherin and clathrin (Fig. 2a). The quantification result also showed that approximately a 3.5-fold increase occurred in the E-cadherin and clathrin interaction in Sh-DDR1 cells compared with that of Mock cells (Fig. 2b). This result indicated that the expression of DDR1 decreases the physical association between E-cadherin and clathrin.

To further confirm that the clathrin-dependent pathway was involved in the knockdown of DDR1-induced E-cadherin endocytosis, we applied a specific clathrin-dependent pathway inhibitor, Dyngo^®^ 4a, to inhibit the activity of dynamin^42^. Because the dissociation of β-catenin and α-catenin decreases membrane stability and increases the turnover rate of E-cadherin^43^, we examined the colocalisation of E-cadherin and β-catenin to determine the junctional localisation and stability of E-cadherin. In Mock cells, E-cadherin and β-catenin formed a sufficient cell-cell adhesion junction regardless of receiving Dyngo treatment (Fig. 2c, upper panels). In Sh-DDR1 cells, E-cadherin and β-catenin did not form a continuous cell-cell junction (Fig. 2c, lower panels, DMSO treatment); treatment with Dyngo^®^ 4a rescued junctional localisation of E-cadherin and β-catenin (Fig. 2c, lower panels) and decreased cytosolic staining of E-cadherin in a dose-dependent manner (Fig. 2c). To quantify the changes of E-cadherin and β-catenin distribution, we analysed the co-localized fluorescence intensity of E-cadherin and β-catenin as well as Pearson’s correlation coefficient using Olympus FV-1000 software. We found that a similar colocalisation pattern of E-cadherin and β-catenin was found in Mock cells regardless of Dyngo treatment. In sh-DDR1 cells, the colocalisation of E-cadherin and β-catenin was significantly reduced and it was significantly rescued by Dyngo treatment (Fig. 2d). To further characterize the correlation of the colocalisation of E-cadherin and β-catenin, we performed the Pearson’s correlation coefficient analysis. There was a similar positive correlation between DMSO and Dyngo in Mock cells. Knockdown of DDR1 significantly decreased the positive correlation of E-cadherin and β-catenin localization whereas Dyngo treatment rescued the knockdown effect (Fig. 2e). In addition, the levels and localisation of clathrin were low and not colocalised with E-cadherin in cells, whereas localisation of clathrin at the cell-cell junctions in DDR1 knockdown M10 cells was increased (Supplemental Fig. 1c). These results suggested that knockdown of DDR1-induced E-cadherin endocytosis is mediated by a clathrin-dependent pathway.

DDR1 decreases the protein dynamic and increases membrane stability of E-cadherin; DDR1-inhibited E-cadherin endocytosis is likely to occur through the clathrin-mediated pathway. However, limited evidence exists to show that the decrease of E-cadherin endocytosis is equivalent to the increase of E-cadherin membrane stability. We performed photoconversion using E-cadherin conjugated with mEos fluorescent protein (HECD-mEosFP)^29^ to examine this hypothesis, after which the emission wavelength of mEos shifted from green to red. The faster the red fluorescence declined and the green fluorescence recovered in the converted area, the more unstable E-cadherin became. Mock and Sh-DDR1 cells were transiently transfected with HECD-mEosFP, treated with DMSO or Dyngo^®^ 4a (80 μM), and photoconverted at the cell-cell junction. Live cell images were taken every 10 min. After photoconversion, Mock cells that had and had not received Dyngo 4a treatment in the 50 min treatment period showed that the intensity of red-HECD-mEosFP was 62.95% and 83.80%, respectively and the intensity of green-HECD-mEosFP remained low (Fig. 2f, control; 2g, left panels). In Sh-DDR1 cells treated with the vehicle, the red-HECD-mEosFP dispersed and the green-HECD-mEosFP shifted into the converted area, which resulted in the relative fluorescence intensity of red-HECD-mEosFP declining to 53.21% and intensity of green-HECD-mEosFP went up to 56.72% within 50 min. After Dyngo treatment, the fluorescence intensity of red-HECD-mEosFP declined to 59.15% within 50 min whereas green-HECD-mEosFP remained low, indicating a rescue effect (Fig. 2f,g, right panels). This result indicated that prevention of clathrin-mediated E-cadherin endocytosis increases localisation of E-cadherin at the cell-cell junction; it can rescue the membrane stability of E-cadherin in Sh-DDR1 cells.

### Knockdown of DDR1 increases Src activity and results in the cytosolic accumulation and instability of E-cadherin

**Figure 3:**
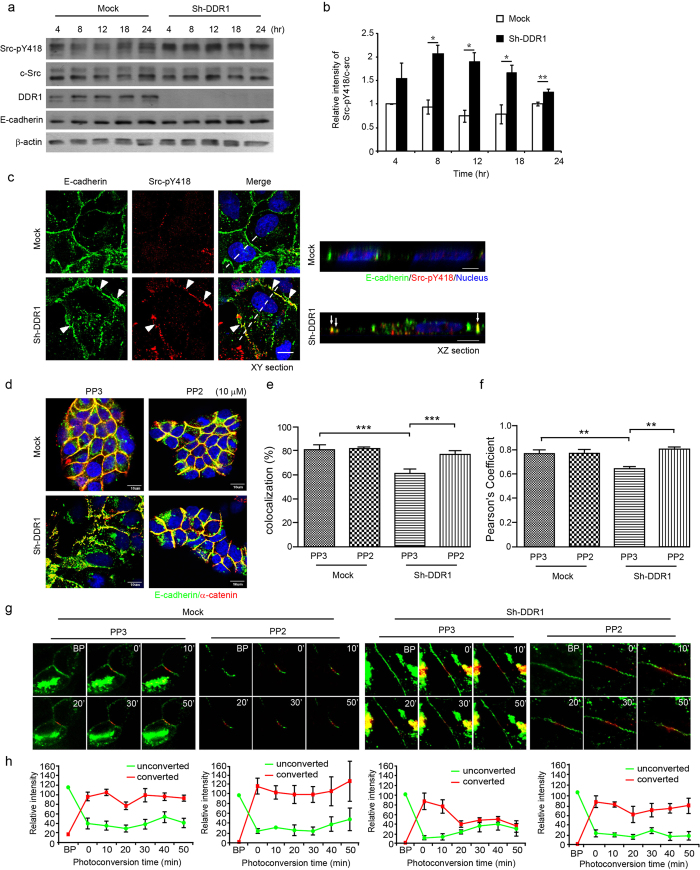
Src mediates knockdown of DDR1-induced E-cadherin endocytosis. (**a**) Mock and Sh-DDR1 cells were seeded on a culture dish at the indicated points of time, and levels of Src-pY418, c-Src, DDR1, and E-cadherin in whole cell lysate were analysed using Western blotting. The quantified result of Src-pY418/c-Src is shown in (**b**). Each bar represents mean ± SE of three independent experiments. *Indicates *P* < 0.05, **indicates *P* < 0.01 to its paired control. (**c**) The localisations of Src-pY418 in Mock and Sh-DDR1 cells were assessed using immunofluorescence staining after cells were cultured for 24 h. The arrow heads mark the junctional colocalisation of E-cadherin and Src-pY418 in the XY section, and the arrows mark the colocalisation of E-cadherin and Src-pY418 in the XZ section. The dashed lines in the XY section indicate the position of the XZ section. Bar: 10 μm. (**d**) Mock and Sh-DDR1 cells were cultured for 24 h and then treated with 10 μM PP3 (control) or PP2 for 4 h. Cells were fixed and immunostained for E-cadherin (green), α-catenin (red), and nuclei (blue). (**e**) The fluorescence intensity profiles of the colocalisation of E-cadherin and β-catenin were assessed using Olympus FV-1000 imaging software. (**f**) The fluorescence intensity profiles of the Pearson’s correlation coefficient of E-cadherin and β-catenin were assessed using Olympus FV-1000 imaging software. **Represents P < 0.01; ***represents P < 0.001. (**g**) Mock and Sh-DDR1 cells were transiently transfected with HECD-mEosFP and incubated on chamber slides for 24 h. After PP3 (10 μΜ) or PP2 (10 μΜ) treatment for 4 h, Mock and Sh-DDR1 cells were subjected to photoconversion analysis and live images were captured using an Olympus FV-100 confocal microscope. Representative fluorescence images of three experiments during the recording time are shown. (**h**) The quantification results of (**g**) are shown as mean ± SE from three independent experiments in parallel with the treatment group.

We sought to identify the signalling pathway for knockdown of DDR1-induced clathrin-mediated endocytosis of E-cadherin. Src activation has been shown to be involved in numerous signalling pathways to induce E-cadherin endocytosis^24^,^37^,^39^. In addition, Src activity negatively regulates cell-cell adhesion; however, it is required for the initiation of adherens junctions^44^. As a result, we proposed that knockdown of DDR1 induces E-cadherin endocytosis through the activation of Src. The levels of Src pY418 in Sh-DDR1 cells were higher than they were in Mock cells (Fig. 1a) after cells were seeded on a plate. By examining the time course for Src pY418 activation after seeding on culture dishes, we found that the levels of Src pY418 were significantly higher after 8 h of seeding and that they were sustained for 24 h (Fig. 3a,b). To examine whether Sh-DDR1-induced Src activation localised at adherens junctions in Sh-DDR1 cells, we applied dual-staining for E-cadherin and Src pY418. In the Mock cells, Src pY418 was barely detectable at cell-cell junctions, whereas in Sh-DDR1 cells, the staining of Src pY418 was increased on the cell periphery and cytosol, as well as on membrane-localised Src colocalised with E-cadherin at cell-cell junctions (Fig. 3c, arrow heads). The XZ section of the dashed line of the merged images indicates that the junctional Src pY418 colocalised with E-cadherin at the lateral membrane, but not in the cytosol (Fig. 3c, XZ section, arrows). These results indicated that knockdown of DDR1 increases Src activity and colocalisation with E-cadherin at cell-cell junctions.

To determine whether Sh-DDR1-induced Src activation mediates E-cadherin endocytosis, we performed Src inhibitor PP2, and PP3 was served as a negative control. In the immunofluorescence staining of Mock cells, E-cadherin and α-catenin were stained at the junctional area regardless of PP2 or PP3 treatment. In Sh-DDR1 cells, PP3 treatment showed discontinuous cell-cell junctions and cytosolic localisation of E-cadherin and α-catenin, whereas PP2 treatment compacted cell colonies and increased junctional E-cadherin and α-catenin staining (Fig. 3d). The colocalisation and Pearson correlation coefficient results of E-cadherin and α-catenin localization also demonstrated that PP2 rescued E-cadherin and α-catenin to the junction (Fig. 3e,f). These results indicate that knockdown of DDR1-induced clathrin-dependent E-cadherin endocytosis is mediated by the activation of Src.

To further clarify whether Src regulates membrane stability of E-cadherin, cells were transfected with HECD-mEosFP and treated with Src inhibitor PP2; live cell images were assessed after photoconversion. No significant effect was shown for PP2 or PP3 in Mock cells before or after photoconversion. In Sh-DDR1 cells receiving PP3 treatment, the fluorescence intensity of red-HECD-mEosFP was reduced to 32.92% within 50 min, and PP2 treatment rescued the relative fluorescence intensity of the red-HECD-mEosFP to 75.19% after 50 min (Fig. 3e). These results indicated that knockdown of DDR1 increases activity of Src to destabilise E-cadherin and cause E-cadherin endocytosis.

### Knockdown of DDR1-increased Src activity is mediated by integrin β1

**Figure 4:**
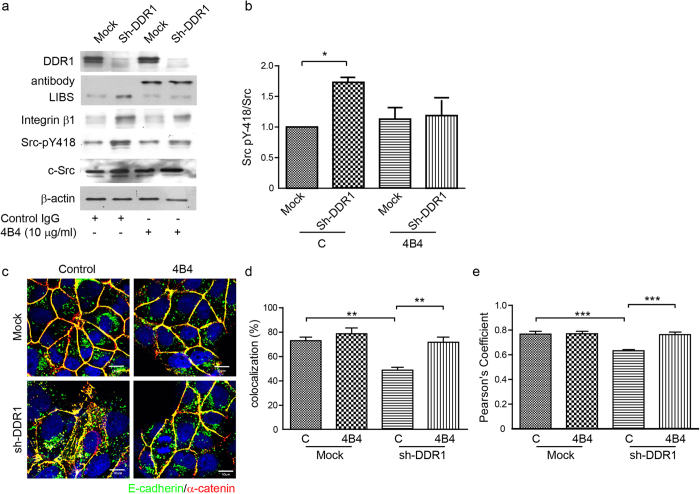
Integrin β1 increases Src activity and endocytosis of E-cadherin. (**a**) Mock and Sh-DDR1 cells were suspended and pretreated with 10 μg/ml control IgG or 4B4 for 30 min, followed by incubation on dishes in normal medium in the absence or presence of 10 μg/ml control IgG or 4B4. After 24 h, total cell lysate was collected and the protein levels were examined using immunoblotting. (**b**) Quantification results of Src-pY418/c-Src are shown in (**a**). Each bar represents mean ± SE of three independent experiments. *Indicates *P* < 0.05 versus Mock control. (**c**) Mock and Sh-DDR1 cells were treated with 10 μg/ml control IgG or 4B4 and plated in chamber slides in the presence of normal medium. After 24 h, cells were fixed and stained for E-cadherin (green), α-catenin (red), and nuclei (blue). (**d**) The fluorescence intensity profiles of the colocalisation of E-cadherin and β-catenin were assessed using Olympus FV-1000 imaging software. (**e**) The fluorescence intensity profiles of the Pearson’s correlation coefficient of E-cadherin and β-catenin were assessed using Olympus FV-1000 imaging software. **Indicates P < 0.01; ***indicates P < 0.001.

Because DDR1 decreased E-cadherin endocytosis through the inhibition of Src activity, we sought to examine how DDR1 regulated Src activity. We proposed that DDR1 decreased Src activity and reduced E-cadherin endocytosis by disrupting integrin signalling. We performed an integrin β1 blocking antibody, 4B4, to block integrin β1 signals^29^,^45^. The flow cytometry results indicated that the 4B4 antibody could bind cell surface integrin β1 of both Mock and Sh-DDR1 cells (Supplementary Fig. 2a). The binding of 4B4 to cell surface integrin β1 suppressed activation of integrin β1, as examined by ligand-induced binding site antibody^46^ (Fig. 4a). In addition, levels of Src pY418 in Sh-DDR1 cells were higher than in Mock cells. After blocking integrin β1 signals, the levels of Src pY418 decreased (Fig. 4a,b), which indicated that Src mediated integrin β1 signalling in Sh-DDR1 cells. We then tested whether inhibition of an integrin β1 signal resulted in an increase in E-cadherin junctional localisation by using double immunostaining of E-cadherin and α-catenin. E-cadherin and α-catenin colocalised at the junctional area and formed a continuous cell-cell junction in Mock cells, regardless of the blocking of integrin β1. Inhibition of the integrin β1 signal decreased cytosolic E-cadherin and increased E-cadherin junctional localisation in Sh-DDR1 cells (Fig. 4c). The colocalisation and Pearson’s correlation analysis also demonstrated that there was no significant change in colocalisation of E-cadherin and α-catenin in Mock cells with or without 4B4 treatment. The colocalisation of E-cadherin and α-catenin was significantly suppressed in sh-DDR1 cells whereas treatment of 4B4 rescued the colocalisation of E-cadherin and α-catenin (Fig. 4d,e). These results indicated that DDR1 prevented E-cadherin endocytosis through disruption of β1 integrin-Src signalling.

### Knockdown of DDR1 increases junctional instability through actin cytoskeleton reorganisation

**Figure 5:**
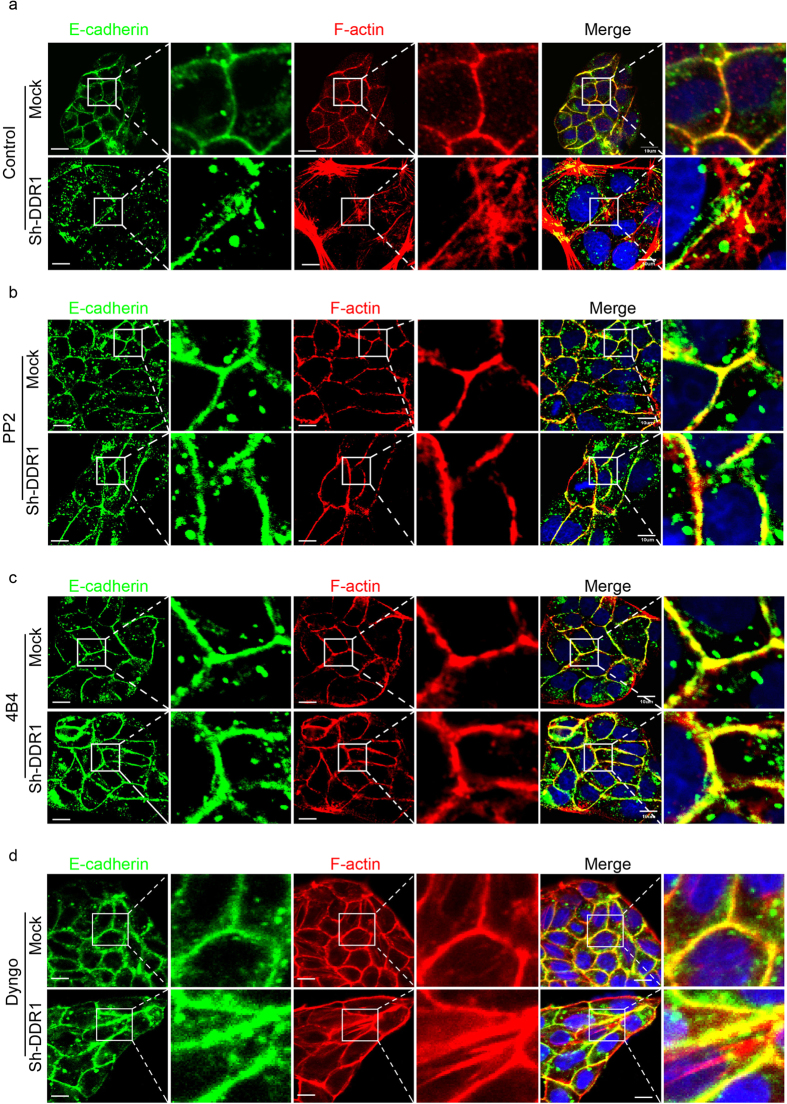
Inhibition of Src and integrin β1, but not clathrin-mediated endocytosis, converts actin cytoskeleton from stress fibres to actin belt. (**a**) Mock and Sh-DDR1 cells were seeded on chamber slides for 24 h, followed by treatment with (**a**) control, (**b**) PP2 (10 μM), (**c**) 4B4 (10 μg/ml), and (**d**) Dyngo (80 μM) for 4 h. After fixation, cells were stained with E-cadherin (green), F-actin (red), and nuclei (blue). The selected squares in the left panels were enlarged and are shown in the right panels. Bar: 10 μm.

The stability of E-cadherin-catenin complex is altered by the reorganisation of actin cytoskeleton. Stress fibre formation disrupts the stability of E-cadherin-catenin-actin complex and increases E-cadherin endocytosis^44^. Therefore, we used immunostaining to compare the actin cytoskeleton organisation of Mock and Sh-DDR1 cells. There was an actin ring around the Mock cells and it colocalised with E-cadherin, whereas there were numerous stress fibres across the Sh-DDR1 cells, which showed only partial colocalisation with E-cadherin (Fig. 5a). Treatment of Src inhibitor PP2 or integrin β1 neutralised antibody 4B4 increased E-cadherin junctional localisation and changed actin cytoskeleton organisation from stress fibres into an actin belt (Fig. 5b,c). In contrast, although Dyngo treatment decreased cytosolic E-cadherin and increased junctional E-cadherin, it did not abolish stress fibre formation in Sh-DDR1 cells (Fig. 5d). These results indicated that inhibition of integrin β1 signalling rescues E-cadherin-catenin-actin complex stability.

**Figure 6:**
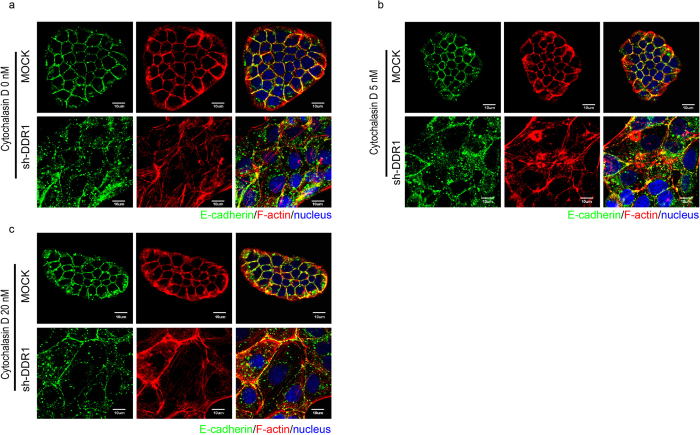
Knockdown of DDR1 increases junctional instability by increasing actin stress fibres. Mock and Sh-DDR1 cells were cultured on chamber slides for 24 h and then treated (**a**) without or with cytochalasin D ((**b**) 5 nM), and ((**c**) 20 nM) for 4 h. Immunostaining was applied to visualise E-cadherin (green), F-actin (red), and nuclei (blue) with specific antibodies. Bar: 10 μm.

Previous study shows that the cadherin–catenin complex directly linked to actin filaments via α-catenin and this cadherin-catenin-actin complex formation is essential for the stability of adherens junction^47^. On the other hand, the discontinuous adherens junctions are found to link with the formation of stress fibres^48^, suggesting that formation of stress fibres may lead to the instability of E-cadherin. We then disrupted stress fibres by treating cells with cytochalasin D (Fig. 6). At 5 nM cytochalasin D treatment, only a minor effect was observed for the rescue of E-cadherin at cell-cell junctions, and the majority of actin continued to form stress fibres (Fig. 6b) compared with the control (Fig. 6a). Treatment with 20 nM cytochalasin D did not affect E-cadherin junctional localisation and actin ring structure in Mock cells. However, E-cadherin produced a linear arrangement at cell-cell junctions in Sh-DDR1 cells; in some cells, actin rings formed and colocalised with E-cadherin (Fig. 6c). At 60 nM cytochalasin D treatment, we observed a possible cytotoxic effect in Mock cells because E-cadherin and F-actin had accumulated abnormally at the cell periphery junction whereas actin rings formed and colocalised with E-cadherin in numerous Sh-DDR1cells (data not shown). These results indicated that knockdown of DDR1 increased integrin β1-Src signalling and the regulation of E-cadherin stability appears to be actin dependent.

### DDR1 Y796-mediated signalling is critical for Src activation and E-cadherin localisation

**Figure 7:**
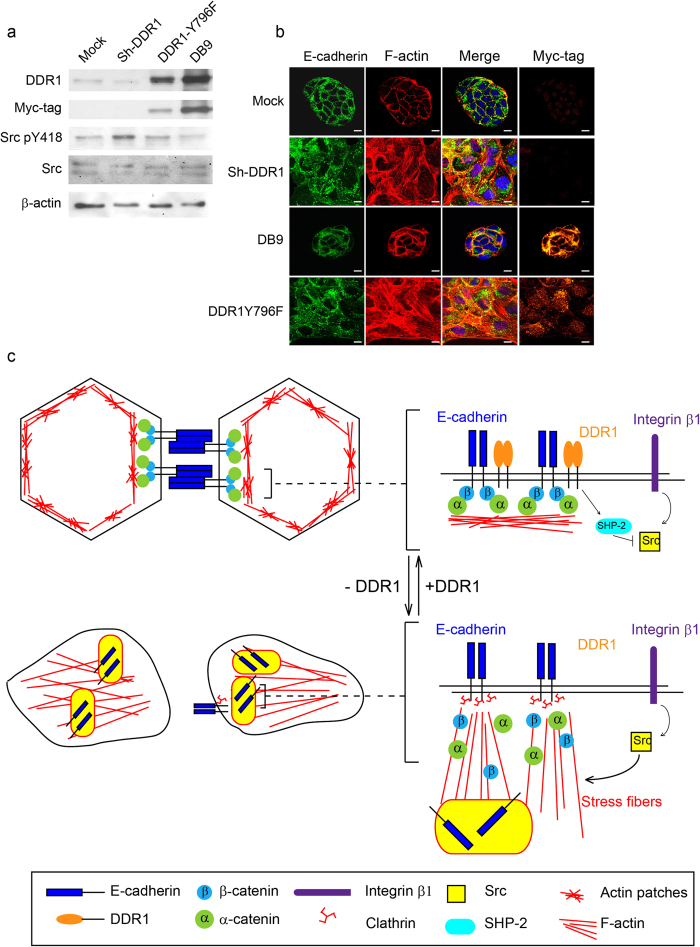
DDR1 Y796-mediated signalling is critical for Src activation and E-cadherin localisation. (**a**) Mock, Sh-DDR1, DDR1 Y796F, and overexpressing cells (DB9) were cultured on culture dishes for 24 h before being lysed. Protein levels of DDR1, myc-tag, Src pY418, and Src were assessed using immunoblotting. (**b**) Mock, Sh-DDR1, DB9, and DDR1 Y796F cells were cultured on chamber slides for 24 h before being fixed. The subcellular localisation of E-cadherin (green), F-actin (red), nuclei (blue), and Myc-tag (orange) were assessed using immunostaining. Bar: 10 μm. (**c**) Proposed model of the current study where DDR1 inhibits Src activity, possibly by activating SHP-2 to maintain E-cadherin stability. In Sh-DDR1 cells, Src activity was elevated, which in turn promoted actin cytoskeleton reorganisation. These reorganised actin stress fibres decreased E-cadherin-catenin complex stability, and the unstable E-cadherins were then recruited through clathrin binding, resulting in E-cadherin endocytosis through the clathrin-mediated pathway.

According to previous studies, DDR1 disrupts integrin β1-induced cell migration through the inhibition of SHP-2 activity^34^. To further study whether DDR1 disrupted integrin β1-Src signalling through the inhibition of SHP-2 activity, we inhibited tyrosine phosphatase activity by using a general tyrosine phosphatase inhibitor, Na_3_VO_4_. Levels of Src pY418 were increased in Mock and Sh-DDR1 cells while using the Na_3_VO_4_ treatment (data not shown). To confirm that DDR1-regulated Src activity may be mediated by the activation of SHP-2, a DDR1 Y796F mutant was applied, which was unable to activate SHP-2^34^. In wild-type DDR1 overexpressing cells (DB9), the level of Src pY418 was low; Y796F expressing cells showed elevated levels of Src pY418 compared with DB9 cells (Fig. 7a). In addition, the immunofluorescence results showed that overexpression of wild-type DDR1 resulted in a more compact morphology than Mock cells; the DDR1 Y796F mutant showed discontinuous cell-cell adhesion and more stress fibres than DB9 clone instead of junctional F-actin, which was similar to the morphology of Sh-DDR1. Because a DDR1 Y796F mutant is unable to bind SHP-2^34^, these results implied that SHP-2 may be recruited by DDR1 pY-796 to suppress Src activity and maintain the junctional stability of E-cadherin.

## Discussion

Our study provided evidence that DDR1 stabilises E-cadherin by inhibiting integrin-Src-mediated endocytosis pathways. However, limited information exists to indicate that DDR1 physically interacts with integrin. It is known that α_2_β_1_ integrin is a major receptor for fibril collagen that binds in MDCK cells and that DDR1 can be activated by Type I, II, III, and IV collagens^31^,^32^. We speculate that DDR1 contends the similar ligands, such as these types of fibril collagens with integrin β1, to regulate E-cadherin membrane stability. In fact, DDR1 and integrin β1 have opposite functions: DDR1 maintains epithelial differentiation and cell polarity^32^, whereas integrin β1 is a mesenchymal marker^49^. When epithelial cells express DDR1, the integrin β1 signal is weakened and results in epithelial differentiation. However, if integrin β1 is expressed and activated, this leads to EMT. Src has direct interaction with the integrin β1 cytoplasmic tail, and integrin clustering stabilises activated Src by inducing intermolecular autophosphorylation^50^. Our study shows that blocking the integrin β1 signal decreases the phosphorylation of Src tyrosine 418 in Sh-DDR1 cells. As a result, the activation of Src in Sh-DDR1 cells may be caused by the competition between DDR1 and integrin β1 signalling. However, Src is also a component of the focal adhesion complex, and the activation of Src promotes an integrin-FAK signalling pathway by enhancing the assembly of dynamic focal adhesion-like structures^44^. This implies that Src and integrin β1 are reciprocally regulated; however, the signal that comes first in our system needs to be further examined.

DDR1 may also regulate Src activity in a non-competitive manner. It has been identified that the extracellular domain of E-cadherin interacts with the extracellular domain of DDR1^36^ and that DDR1 interacts and augments the phosphatase activity of SHP-2 through tyrosine residues 703 and 796^34^. It has also been proved that SHP-2 activates Src through dephosphorylation of Src at tyrosine residue 529^51^. However, DDR1 activity is blocked by its interaction with E-cadherin^52^, which decreases Src activity through the inhibition of SHP-2 activity and may play a role in stabilising E-cadherin on cell membranes. Although the physical interaction of DDR1 and E-cadherin may be required to suppress E-cadherin endocytosis, further experimentation is required.

In addition, Src regulates actin cytoskeleton organisation through numerous pathways, one of which is mediated by integrin/FAK signalling. In colon cancer cells, overexpression of constitutively activated Src stimulates integrin/FAK signalling, which results in lamellipodial ruffling through ERK/MLCK/myosin pathways^44^. A second pathway is mediated by phospholipase Cγ1 (PLCγ1) and Rho GTPase Cdc42/Rac1. PLCγ1 modulates integrin-mediated cell spreading and plays a key role in integrin-dependent cell motility^51^. Pax-interactive exchange factor (β-Pix)/G protein-coupled receptor kinase interacting protein 1 (GIT1)/PLCγ1 complex proteins are the upstream regulators that activate Cdc42 and Rac1 to modulate actin reorganisation. β-Pix is a dual-function guanine exchange factor (GEF)/signalling-effector for Cdc42 and Rac1^53^,^54^. Src activity is required for PLCγ1 to activate calpain, which acts with β-Pix to activate Cdc42/Rac1^51^. In addition, the association of PLCγ1 with GIT1/β-Pix complexes is essential for the function of PLCγ1 for cell-spreading^51^. We therefore speculated that the activation of Src may activate Cdc42/Rac1 through β-Pix/GIT1/PLCγ1 complexes and promote actin reorganisation. DDR1 can also inhibit integrin-mediated cell spreading^35^ and reduce cytosolic E-cadherin through inactivation of Cdc42^29^. Therefore, activated Src in Sh-DDR1 cells likely promotes actin reorganisation through the Cdc42 pathway.

Stress fibre formation decreases E-cadherin-catenin-actin complex stability. A previous study showed that two actin pools regulated the stability and mobility of homo-E-cadherin clusters^28^: actin patches (i.e., the structure to which actin concentrates in cell-cell junctions and which stabilises homo-E-cadherin) and an actin network. These two pools possess different functions and are intermingled at adherent junctions. In the present study, Mock cells presented a stable structure of actin patches (Fig. 5). After blocking of integrin β1-Src signalling, Sh-DDR1 cells also showed a structure of actin patches. This observation suggested that DDR1 stabilises E-cadherin through the reorganisation of actin filaments. This circulated and patched structure may provide a physical linkage between E-cadherin and an actin ring on the cell membrane. Previous studies have shown that actin stress fibres provided a tensile force that tethered E-cadherin into cytosol and that cutting the stress fibres by using laser-irradiation resulted in the accumulation of E-cadherin at cell-cell junctions^55^,^56^. This stress fibre-regulated E-cadherin cytosolic localisation requires ROCK1 to generate tensile force around the cells at the apical junctions, and blocking ROCK1 activity promoted stress fibre formation^57^. In this study, we observed that stress fibres in Sh-DDR1 cells formed perpendicularly to cell-cell junctions. At these sites, E-cadherin was parallel to the stress fibres, indicating that it was tethered by the stress fibres (Fig. 5). If DDR1 regulates E-cadherin stability through an actin cytoskeleton, disrupting the stress fibres of Sh-DDR1 cells may rescue E-cadherin junctional localisation.

E-cadherin may also regulate actin cytoskeleton organisation. E-cadherin may function as a mechanosensor that modulates the actin cytoskeleton in response to applied force by using the actin binding proteins α-catenin and vinculin^58^. The force sensing ability of an adherens junction, which changes its stiffness in response to shear stress, is abolished when blocking the function of E-cadherin^58^. As a result, E-cadherin and the actin cytoskeleton may play reciprocal roles in regulating the turnover of adherens junctions.

In adherens junctions, α-catenin does not directly bind to the actin cytoskeleton. Instead, it binds to the actin cytoskeleton through various actin-binding proteins, such as vinculin, ZO-1, afadin, and Eplin^57^. Vinculin is also a component of the focal adhesion complex; its localisation at focal adhesion sites or at adherens junction sites regulates the stability of E-cadherin^59^. A previous study demonstrated that DDR1 expression decreases the focal adhesion complex^35^, which suggests that DDR1 may promote localisation of vinculin at adherens junctions by disrupting the focal adhesion complex. In addition, knockdown of vinculin and of Eplin disrupted E-cadherin junctional localisation^57^. In Eplin knockdown cells, stress fibres and E-cadherin are arranged vertically with cell-cell junctions. Expression of α-catenin-Eplin fusion protein results in E-cadherin junction accumulation^57^. Therefore, the binding affinity of vinculin and Eplin on the adherens junction may be useful for the maintenance of DDR1-regulated E-cadherin membrane stability.

Although it has been proved that DDR1 promotes cell differentiation and maintains epithelial polarity^36^, numerous studies have indicated that DDR1 is overexpressed in cancer cells and that this overexpression is positively correlated with the migration ability of cancer cells and disease progression^60^. This can be explained by the concept of collective migration. Cancer cells exhibit two types of migration strategies: single cell migration and collective migration. Mesenchymal-like cells exhibit poor cell-cell contact and prefer migrating as a single cell. This type of migrating cells move faster than its normal counterpart and favours metastases through blood vessels. However, cells that do not perform an epithelial-mesnchymal transition maintain a high level of E-cadherin, thus maintaining the integrity of adherens junctions and moving as a cohort. This type of migration typically occurs in local invasion and lymphatic metastasis^61^. A previous study demonstrated that DDR1 inhibits myosin light chain phosphorylation at cell-cell contact and decreases the traction forces that pull cells apart, thus causing them to migrate in a collective manner^62^. In contrast, knockdown of DDR1 resulted in the actomyosin force being generated at cell-cell contacts, the traction force pulling cells apart and thus causing them to move as single cells^62^. These studies show that DDR1 may be expressed in non-EMT cancer cells, stabilising E-cadherin at cell-cell junctions, and promoting collective migration. DDR1 may be down-regulated in certain types of cancers, thus promoting focal adhesion and stress fibre formation, and resulting in the endocytosis of E-cadherin.

## Conclusion

This study demonstrates that DDR1 regulates E-cadherin junctional stability through the inhibition of integrin β1-Src signalling-mediated actin stress fibre formation and clatherin-mediated endocytosis (Fig. 7c).

## Methods

### Materials

pSM2 vector expressing small hairpin RNA against *Canis lupus familiaris* DDR1 was purchased from GenDiscovery Biotechnology^35^. pcDNA3 expressing vector-encoding human E-cadherin (HECD) was obtained as a gift from Dr Barry M. Gumbiner (Memorial Sloan-Kettering Cancer Center, New York, USA). The p221-Ecadh-mEosFP plasmid provided by Dr. Thomas Lecuit was reconstructed as previously described^35^.

Polyclonal anti-DDR1, anti-α-catenin, and monoclonal anti-c-Src antibodies were purchased from Santa Cruz Biotechnology Inc. (Dallas, TX, USA). Monoclonal anti-E-cadherin, anti-β-catenin, and anti-integrin β1 antibodies were purchased from BD Biosciences (San Jose, CA, USA). Monoclonal anti-β-actin antibody was purchased from Novus Biologicals (Littleton, CO, USA). Polyclonal anti-EEA-1 and anti-clathrin heavy chain antibodies were purchased from Abcam (Cambridge, MA, USA). Monoclonal anti-E-cadherin antibody (rat IgG1 isotype) was purchased from Sigma-Aldrich (St Louis, MO, USA). Polyclonal anti-Src pY418 antibody was purchased from ThermoFisher Sci-Invitrogen (Grand Island, NY, USA). Anti-integrin β1 blocking antibody, 4B4, was purchased from Beckman Coulter (Indianapolis, IN, USA). Small interfering RNA (siRNA) specifically binding to a human DDR1 mRNA sequence (ONTARGETplus siRNA pool) was purchased from GE Dharmacon (Lafayette, CO, USA).

### Cell culture

Mardin-Darby canine kidney (MDCK) cells (purchased from ATCC, Manassas, VA, USA) transfected with either control vector (Mock) or DDR1 small hairpin RNA (Sh-DDR1) were cultured in Dulbecco’s modified Eagle’s medium and supplemented with 10% fetal bovine serum (FBS), 100 IU/ml penicillin, and 100 μg/ml streptomycin (all purchased from ThermoFisher Sci-Invitrogen, Grand Island, NY, USA). Sh-DDR1 stable clones were selected using puromycin (Sigma-Aldrich, St. Louis, MO, USA) at 1 μg/ml. The immortalised human pancreatic duct epithelial cell line (HPDE)^63^ and mammary gland epithelial cell line (M10) were provided by Dr Yan-Shen Shan and Dr Ming-Der Lai, respectively (National Cheng Kung University). The HPDE cells were maintained in a keratinocyte serum free medium, which contained human recombinant epithelial growth factor (rhEGF 1–53) and bovine pituitary extract. M10 cells were maintained in α-MEM and supplemented with 10% FBS. All cells were maintained at 37 °C in humidified air containing 5% CO_2_.

### Immunoblotting and immunoprecipitation

Cells were lysed using a RIPA buffer containing 50 mM Tris-HCL, 150 mM NaCl, 1% Nonidet P-40, 0.5% sodium deoxycholate, 0.1% SDS, 4 mM sodium orthovanadate, 200 μM PMSF, and Complete^TM^ (Roche, Basel, Switzerland). The homogenate was stored at -80 °C prior to analysis. Protein concentration was measured using the method demonstrated by Lowry *et al*.^64^, and bovine serum albumin was used as the standard. For immunoblotting, 20 μg of total proteins were resolved using sodium dodecyl sulfate-polyacrylamide gel electrophoresis and electrophoretically blotted onto polyvinylidene fluoride (PVDF) paper (Thermo Scientific, Waltham, MA, USA). The PVDF paper was incubated with primary antibody and the immunocomplexes were detected with horseradish peroxidase-conjugated IgG. The final immunocoplexes were made visible using fluorography with an enhanced chemiluminescence reagent (GE Healthcare Life Sciences, Uppsala, Sweden).

For immunoprecipitation, 1 mg of protein lysate was incubated overnight at 4 °C with 1 μg of primary antibody against E-cadherin. After incubation with protein G-sepharose beads (GE Healthcare Life Sciences, Uppsala, Sweden), the immunocomplex was resolved using immunoblotting.

### Endocytosis assay

This method was a modification on the biotinylation method, which was described by Arancibia-Cárcamo *et al*.^40^. After seeding on chamber slides for 24 h, Mock and Sh-DDR1 cells were rinsed with phosphate buffer saline containing 0.5 mM Mg^2+^ and 1 mM Ca^2+^. Cells were subsequently incubated with 0.1 mg/ml EZ-link NHS-SS-biotin (Thermo Scientific, Waltham, MA, USA) in phosphate buffered saline (PBS) at 4 °C with gentle rocking for 12 min. After rinsing with PBS containing Mg^2+^ and Ca^2+^, cells were rinsed with a quenching buffer (50 mM glycine and 0.5% BSA dissolved in PBS) three times at 4 °C, and then incubated in a 37 °C incubator with normal media for 30 min. After being removed from the media, cells were incubated with a reducing buffer (50 mM glutathione, 75 mM NaCl, 10 mM EDTA, 75 mM NaOH, and pH 7.5–8.0). Finally, the cells were fixed and the immunofluorescence method was applied for E-cadherin, EEA-1, and biotin. EZ-link NHS-SS-biotin was recognised using Neutroavidin-alexa 594 antibody (ThermoFisher Sci.-Invitrogen, Grand Island, NY, USA). Images were taken using an Olympus FV-1000 confocal microscope (Olympus, Tokyo, Japan).

### Treatment of inhibitors

After being cultured for 24 h, cells were treated with a specific dynamin inhibitor Dyngo^®^ 4a^42^ (Abcam, Cambridge, UK). We also performed Src inhibitor PP2 (4-amino-5-(4-chlorophenyl)-7-(*t*-butyl)pyrazolo[3,4-*d*]pyramidine) (10 μM) and PP3 (4-amino-1-phenylpyrazolo[3,4-*d*]pyrimidine) (10 μM) (both purchased from Biovision, Milpitas, CA, USA) by using the same concentration that was used as a negative control of PP2. Cells were treated with various inhibitors for 4 h before we captured live images or collected cell lysates. To block integrin β1 signalling, we performed the blocking antibody 4B4 (10 μg/ml) (EMD Millipore, Billerica, MA, USA) to treat Mock and Sh-DDR1 cells after they were trypsinised; 4B4 was added to suspended cells and incubated for 30 min. The suspended cells were then seeded on culture dishes in the presence of 10 μg/ml 4B4 for 24 h, after which time the cells were collected for immunoblotting or immunofluorescence.

### Immunofluorescence

Mock and Sh-DDR1 cells were seeded on chamber slides for 24 h and then fixed with 4% para-formaldehyde (Merck, Whitehouse Station, NJ, USA) in PBS for 5 min at room temperature. The fixed cells were rinsed with PBS and permeabilised with 0.25% Triton X-100 in PBS for 10 min. Cells were then soaked in SuperBlock^®^ blocking buffer (Thermo Scientific, Waltham, MA, USA) for 1 h at room temperature, followed by incubation with primary antibody over night at 4 °C. After being rinsed with PBS-Tween 20, the cells were incubated with either Alexa 488 or Alexa 594-conjugated antibody and Hoechst 33342 for 1 h at room temperature. In some cases, combination of Alexa 488 and Rhodamine Phalloidin and Hoechst 33342 (all purchased from ThermoFisher Scientific-Invitrogen, Grand Island, NY, USA) was used to stain E-cadherin and F-actin. The fluorescence images were captured using an Olympus FV-1000 confocal microscope.

### Transient transfection

Mock and Sh-DDR1 cells were seeded at 5 × 10^5^ in a 6-cm dish for 16 h before transfection. Four micrograms of plasmid DNA or 10 nM siRNA were dissolved in 100 μl Opti-medium (ThermoFisher Sci-Invitrogen, Grand Island, NY, USA) and mixed with 20 μl LipofectAMINE^TM^ 2000 (ThermoFisher Sci-Invitrogen, Grand Island, NY, USA). The complex was then added to the cells with normal medium containing 10% fetal calf serum and incubated for 4 h at 37 °C. The transiently transfected cells were trypsinised, replated on glass-bottomed slide dishes, and incubated for 24 h before receiving chemical treatment and processing so that images could be produced.

### Photoconversion

After transient transfection of HECD-mEosFP, Mock and Sh-DDR1 cells were treated with chemical inhibitors for 4 h. A healthy and high level of HECD-mEosFP expressing cells were selected for photoconversion. Laser beams 405 nm in length with 0.5% laser output intensity were used for 10 s to convert the fluorescence. The converted area was approximately 2 μm at cell-cell junctions. After photoconversion, live cell images were taken using a confocal microscope (Olympus FV-1000) every 10 min. The changes in fluorescence intensity were recorded and calculated using FV-1000 software.

### Statistical analysis

The Western blotting results were quantified using ImageJ software. All data are expressed as mean ± standard error of mean (SE) for at least three independent experiments. Student’s *t* test or one-way analysis of variance was used to test for statistical differences by using GraphPad Prizm Version 5.0. Turkey’s procedure was used to test the differences between individual treatment groups. Statistical significance was set at *P* < 0.05.

## Additional Information

**How to cite this article**: Chen, H.-R. *et al*. DDR1 promotes E-cadherin stability via inhibition of integrin-β1-Src activation-mediated E-cadherin endocytosis. *Sci. Rep*. **6**, 36336; doi: 10.1038/srep36336 (2016).

**Publisher’s note:** Springer Nature remains neutral with regard to jurisdictional claims in published maps and institutional affiliations.

## Supplementary Material

Supplementary Information
