# Supplementary material for: DDR1 promotes E-cadherin stability via inhibition of integrin-β1-Src activation-mediated E-cadherin endocytosis
Source: Sci Rep. 2016 Nov 8;6:36336. doi: 10.1038/srep36336 (PMC5099905; doi:10.1038/srep36336)
Supplement: Supplementary Information [file srep36336-s1.doc]

DDR1 promotes E-cadherin stability via inhibition of integrin-β1-Src activation-mediated E-cadherin endocytosis

Hong-Ru Chen^1, #^, Yi-Chun Yeh^1, #, †^, Ching-Yi Liu^1,2^, Yu-Ting Wu^3^, Fang-Yu Lo^3^, Ming-Jer Tang^1,2,*^ and Yang-Kao Wang^2, 3,*^

^1^Department of Physiology and ^2^Institute of Basic Medical Sciences, ^3^Department of Cell Biology and Anatomy, College of Medicine, National Cheng Kung University, Tainan, Taiwan

^†^Current address: Department of Physiology, Anatomy and Genetics, University of Oxford, Oxford, United Kingdom.

*Correspondence should be addressed to Drs. Ming-Jer Tang, MD, PhD ([mjtang1@mail.ncku.edu.tw](mailto:mjtang1@mail.ncku.edu.tw), Tel: 886-6-2353535 ext 5425) and Yang-Kao Wang, PhD ([humwang@mail.ncku.edu.tw](mailto:humwang@mail.ncku.edu.tw), Tel: 886-6-2353535 ext 5333)

#: These authors contributed equally to the paper.

Running title: DDR1 inhibits E-cadherin endocytosis


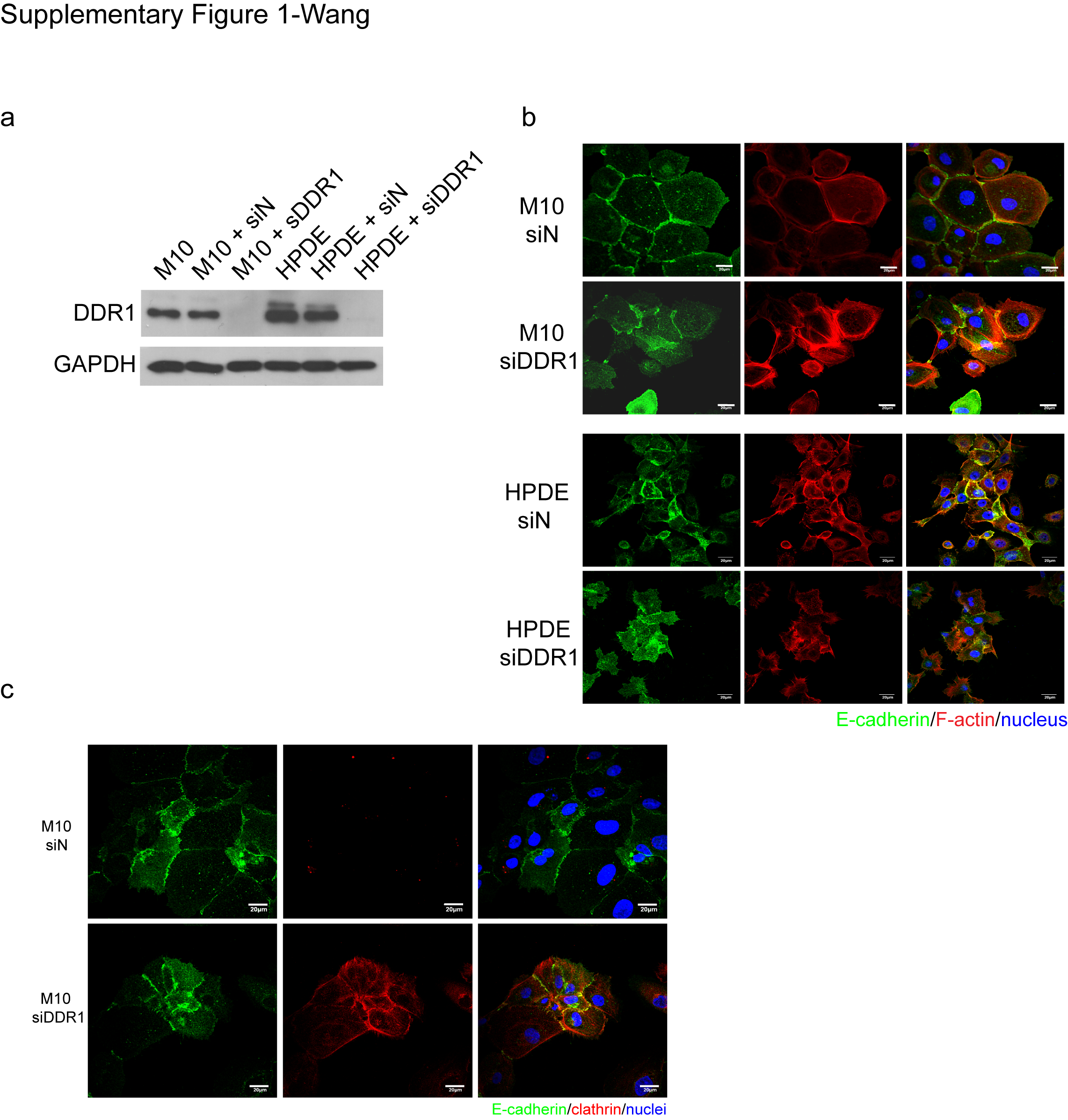


**Figure S1. Knockdown of DDR1 decreases junctional localization of E-cadherin in epithelial cell lines.** Immortalized human mammary epithelial cells M10 or pancreatic duct epithelial cell HPDE were transfected with scramble siRNA (siN) or siDDR1. Cells were then trypsinized, re-plated on tissue culture dish or chamber slide for 24 hrs. (A) The cell lysate was harvested and the levels of DDR1 were analyzed by immunoblotting. (B) Cells were fixed and stained with E-cadherin (green), F-actin (red) and nuclei (blue). Bar: 20 μm. (C) M10 cells transfected with scramble siRNA (siN) or siDDR1 and cells were fixed and stained with E-cadherin (green), clathrin (red) and nuclei (blue). Bar: 20 μm.


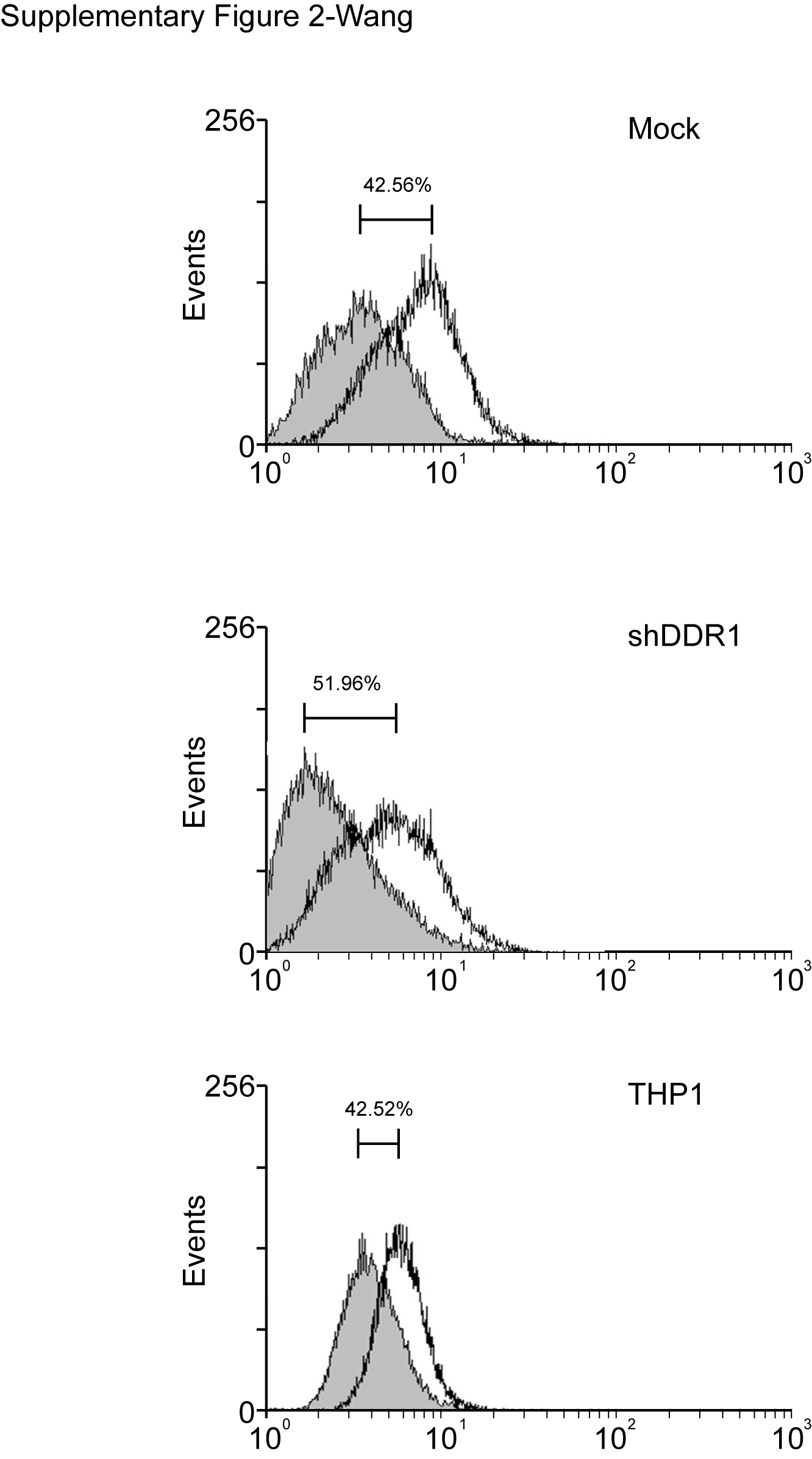


**Figure S2**. **Binding of 4B4 antibody to MDCK cells.** Mock and Sh-DDR1 cells were trypsinized and then incubated with control IgG or 4B4 antibody (10 μg/ml) for 1 hr, followed by incubated with secondary antibody-conjugated with FITC. Cells were then fixed by 1% paraformaldehyde and the binding of 4B4 to cells was analyzed by FACSCalibur (BD Biosciences, San Jose, CA, USA). Human THP-1 cells were performed as a positive control.

Western blots for Figures


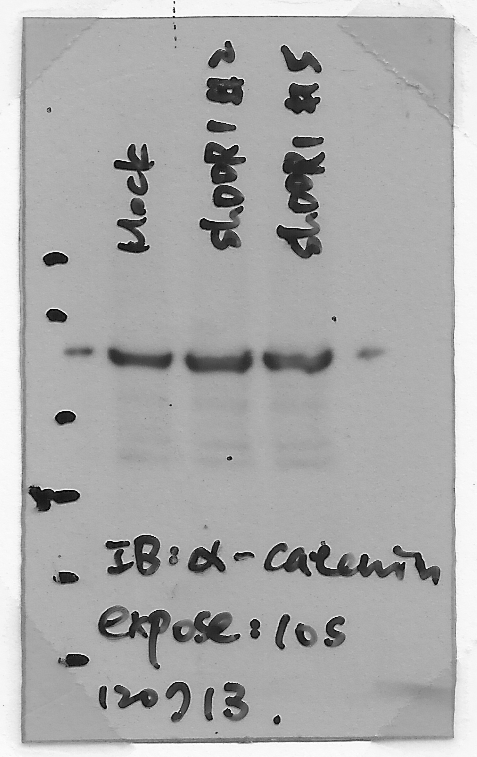

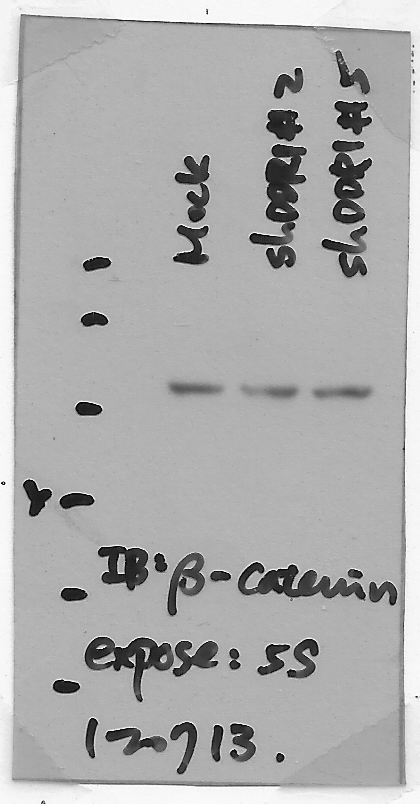

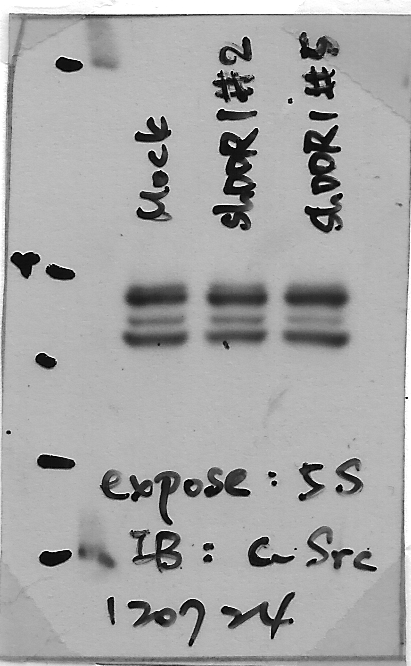

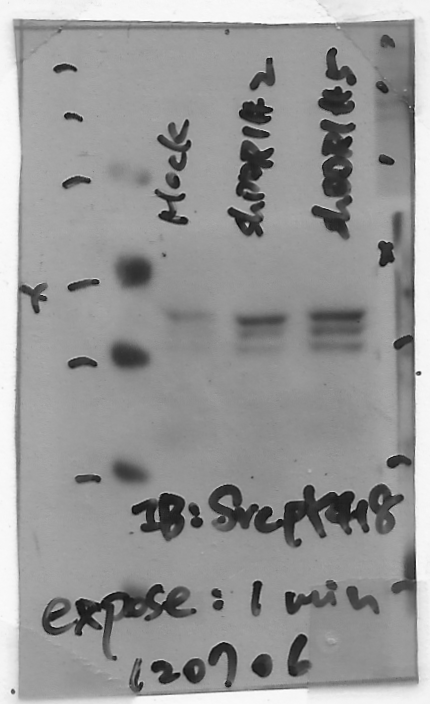

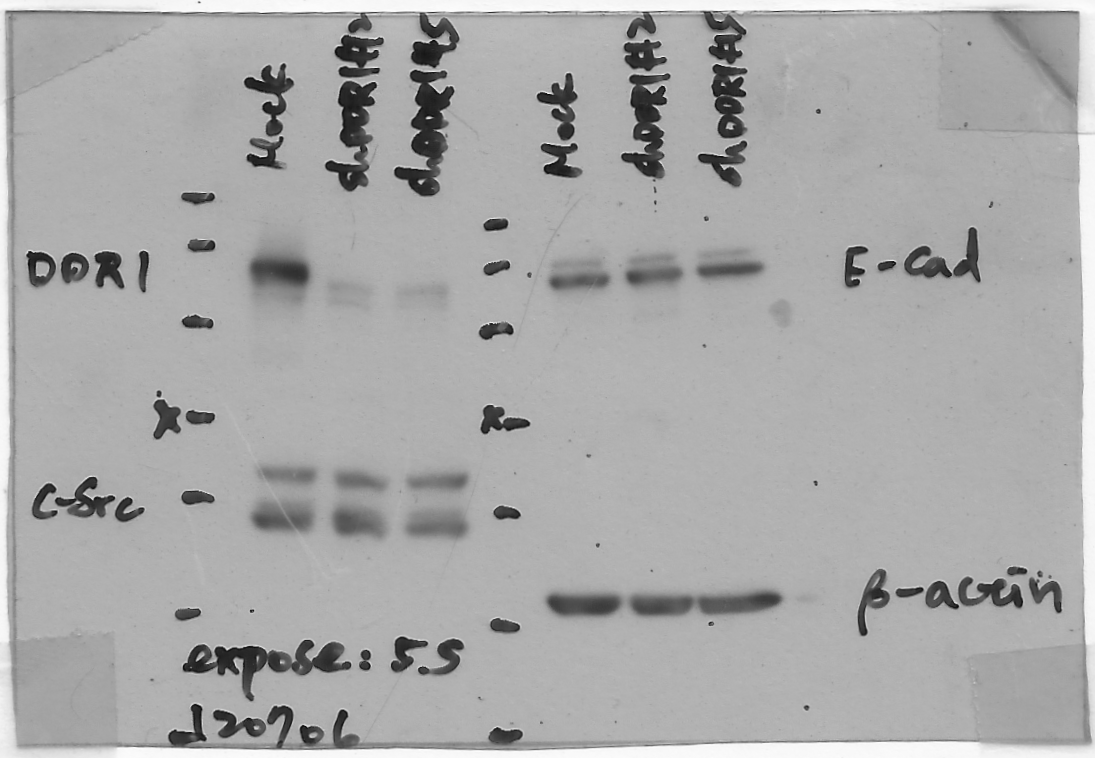


Figure S3. Western blot results for Figure 1


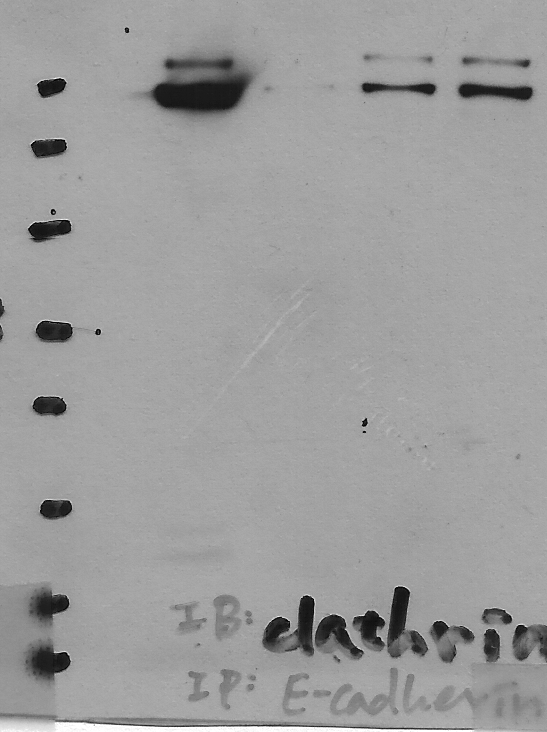

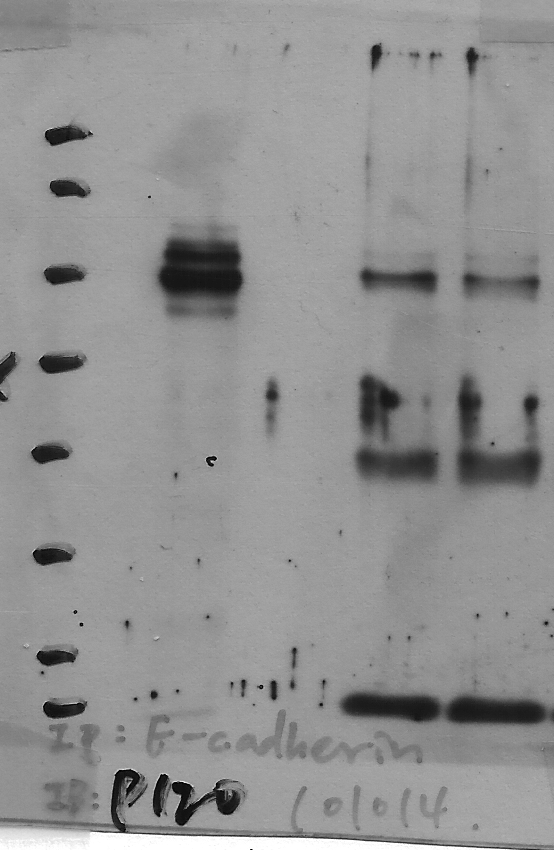


Figure S4. Western blot results for Figure 2.


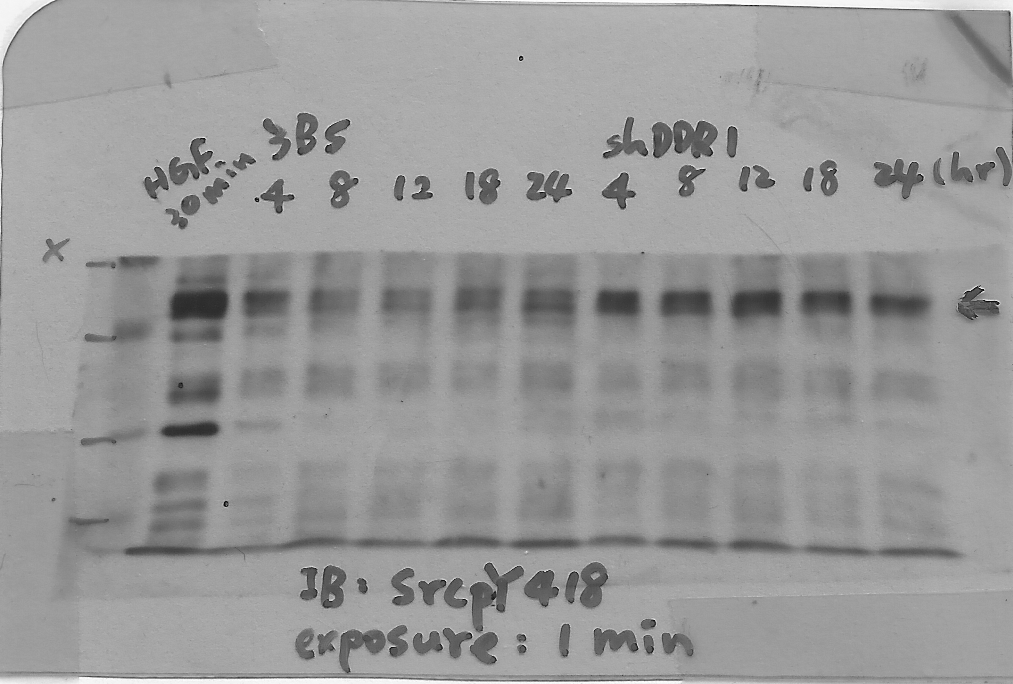

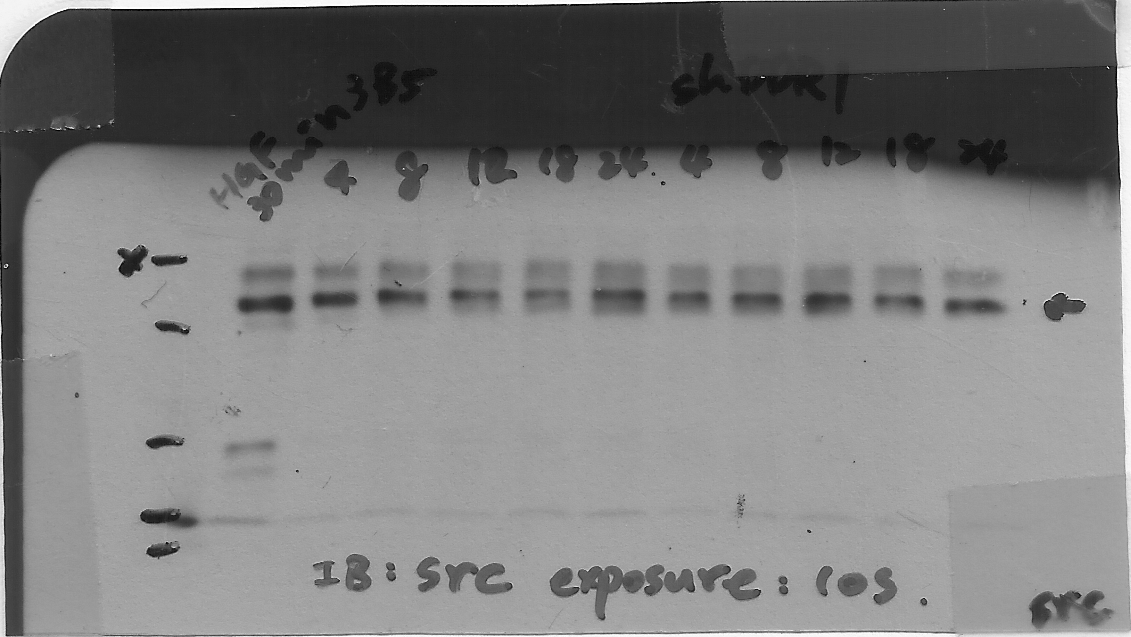


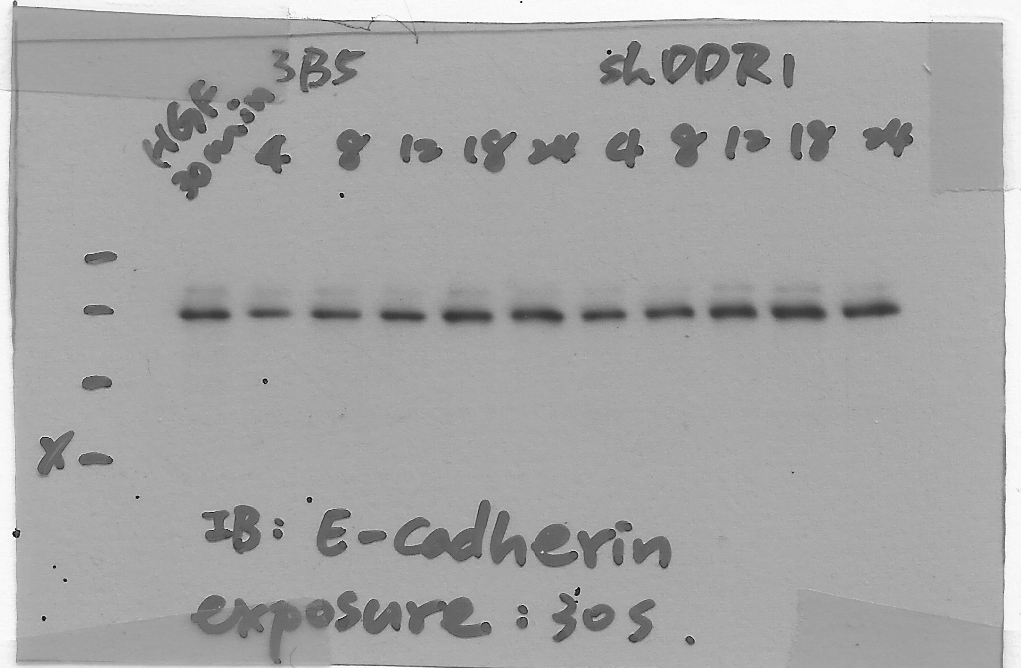

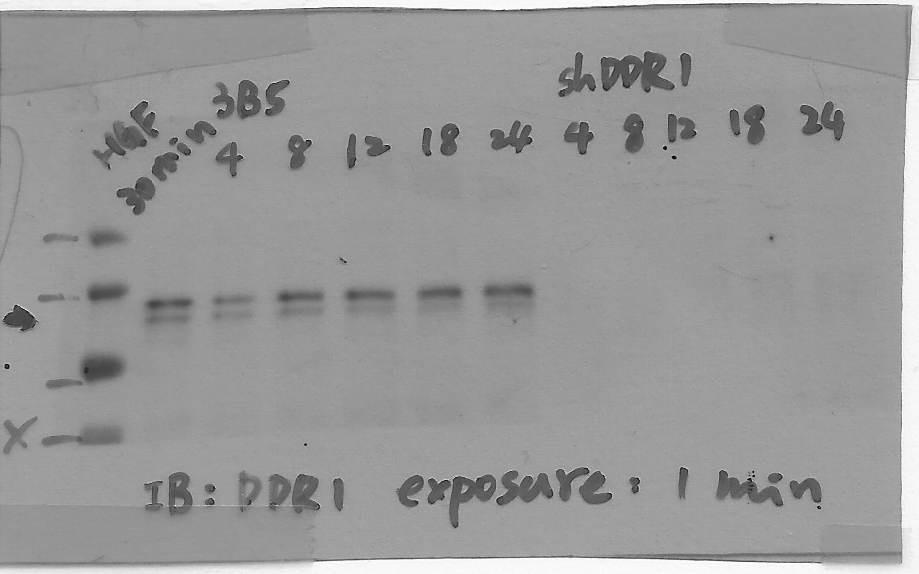


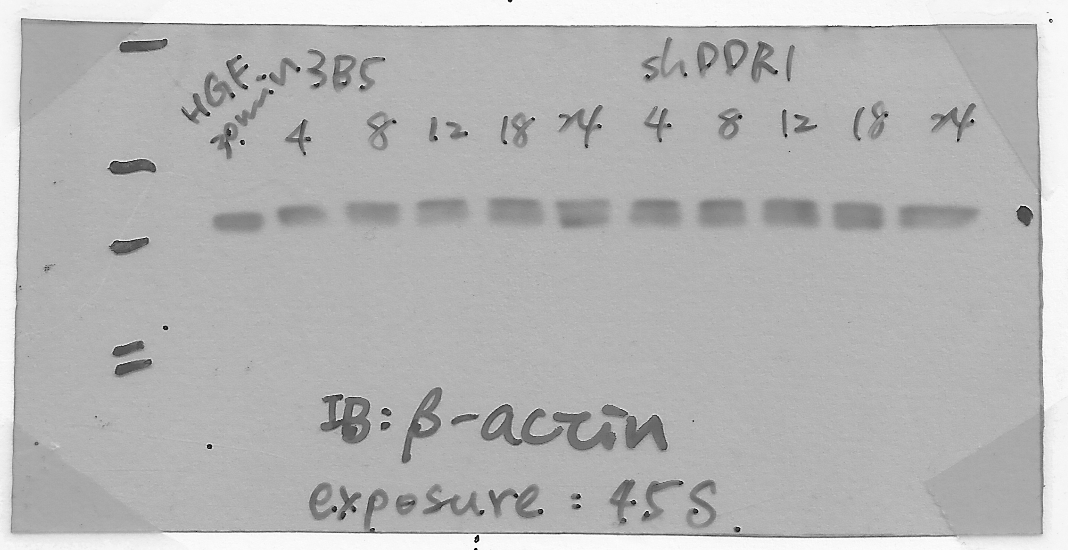


Figure S5. Western blot results for Figure 3.


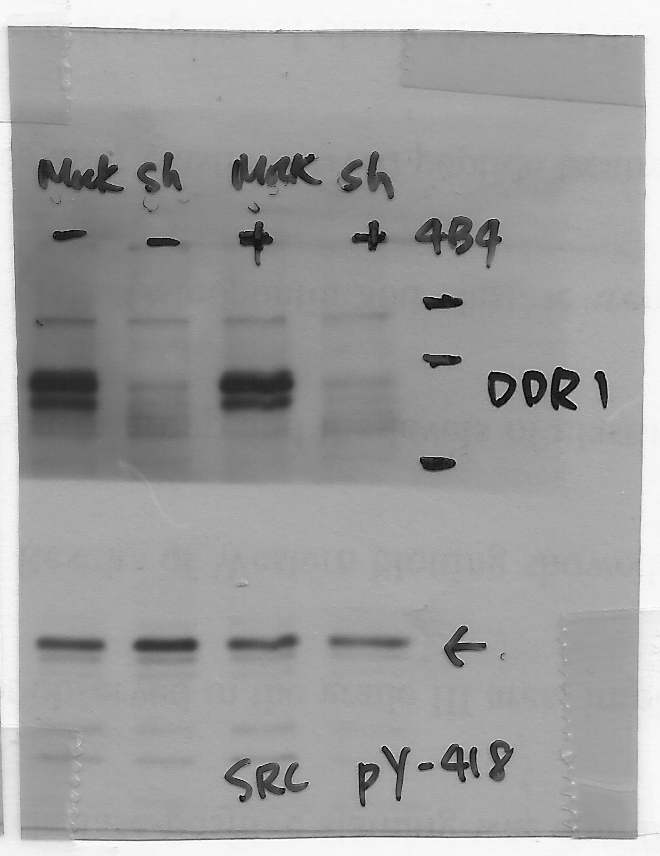

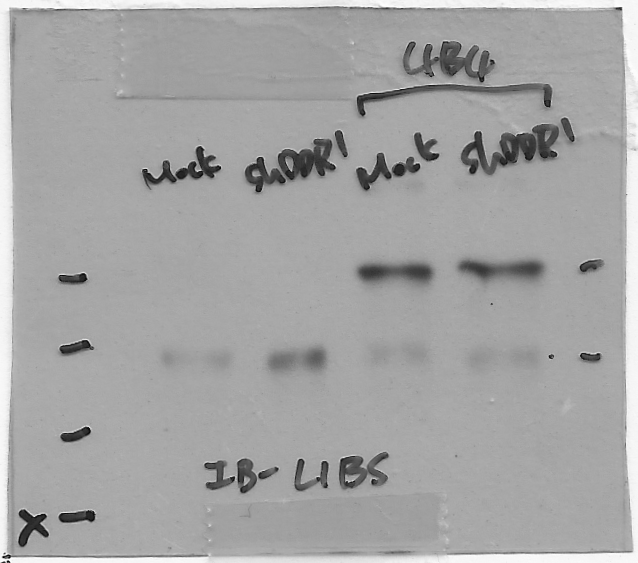


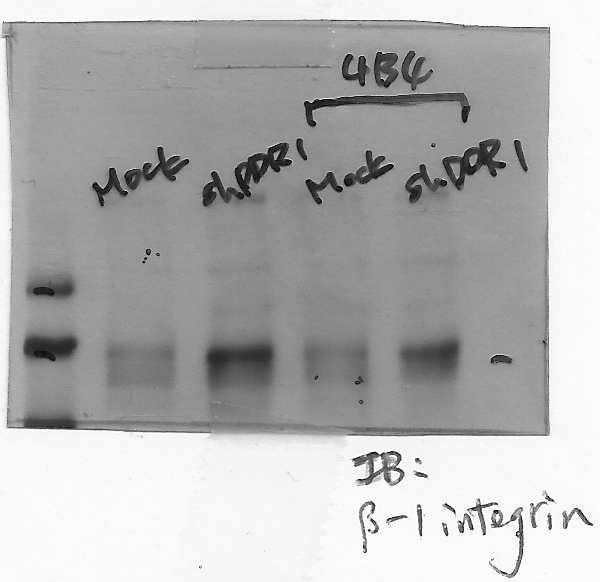

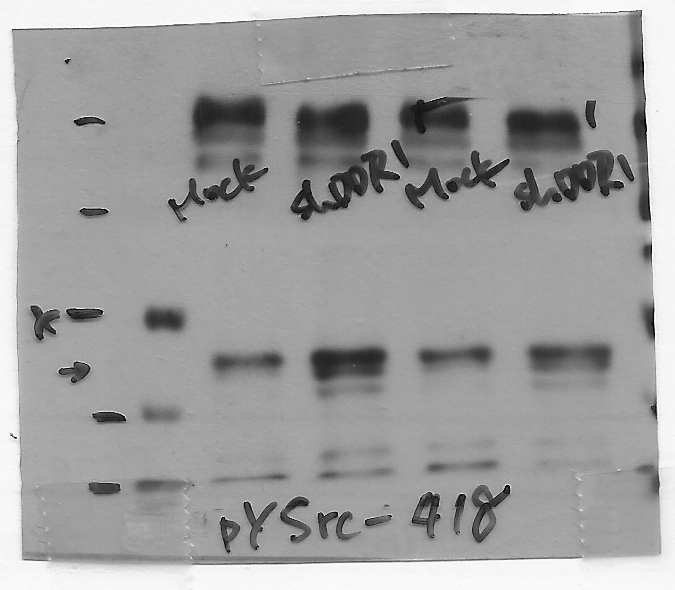


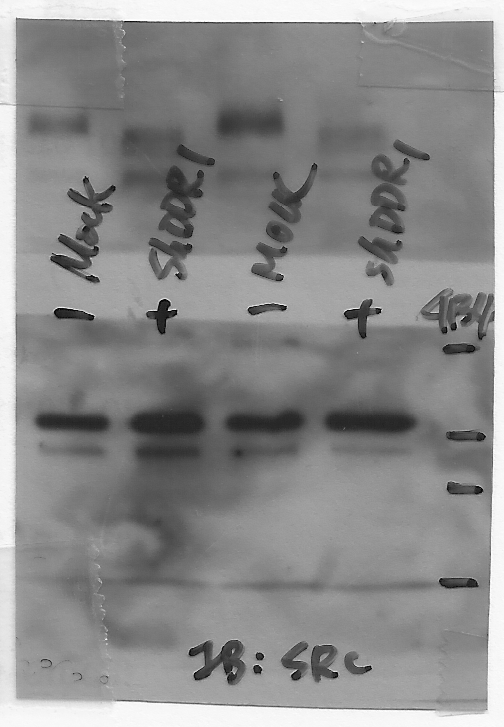

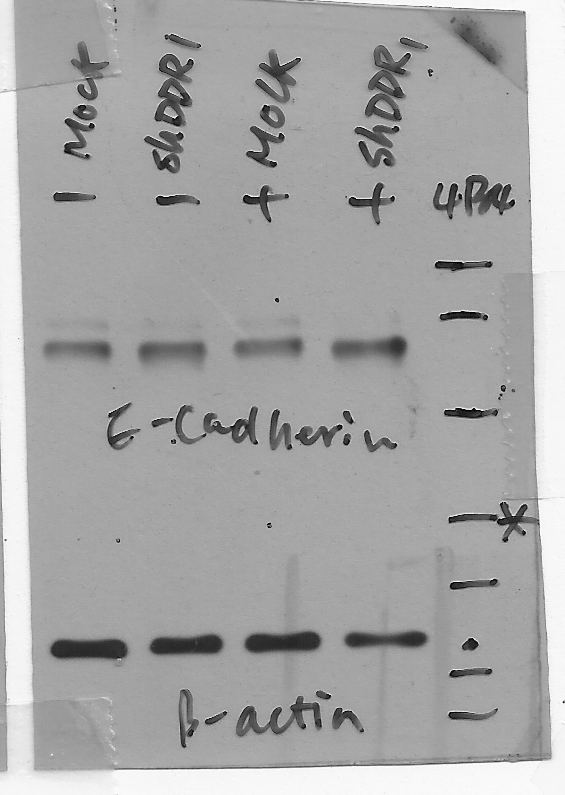


Figure S6. Western blot results for Figure 4.


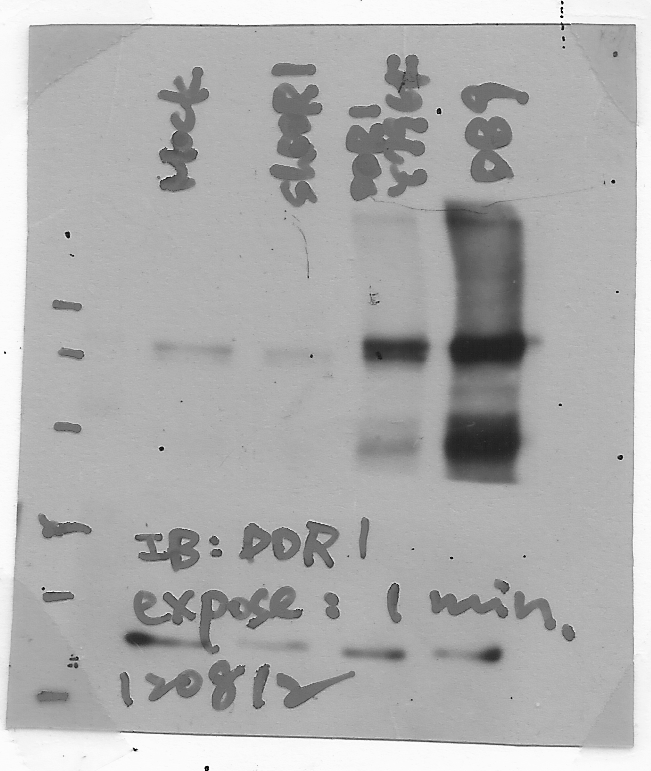

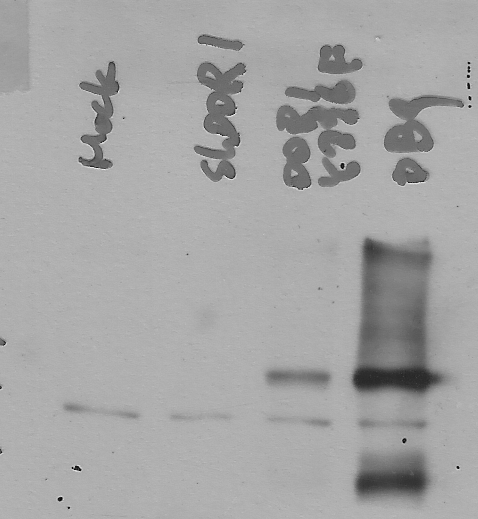

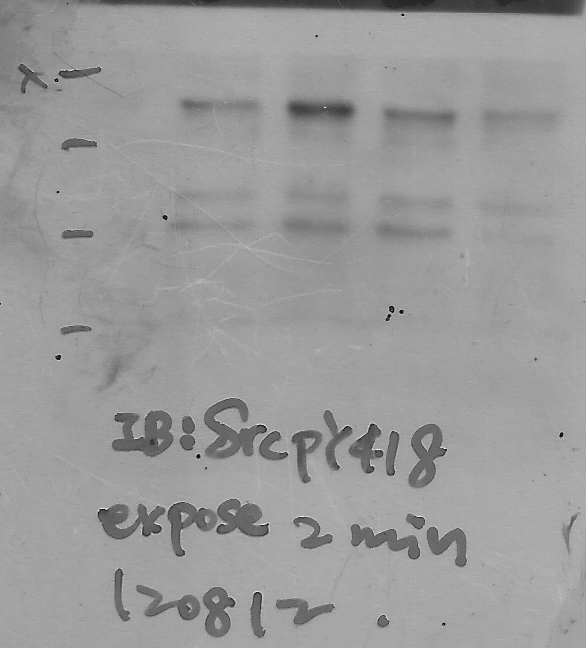


Anti-c-myc


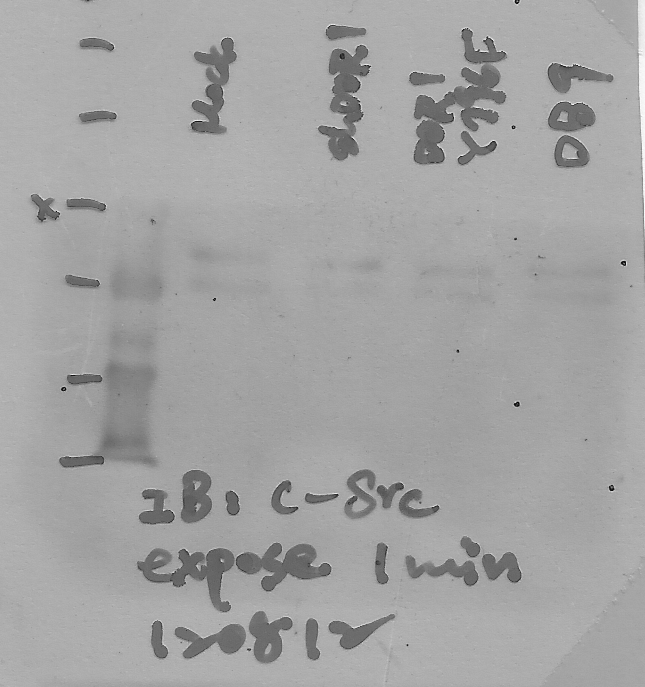

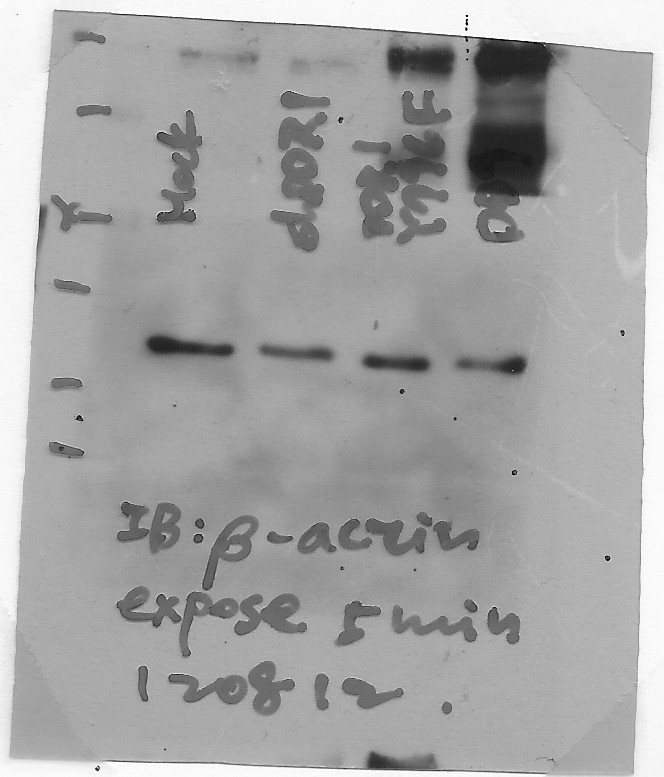


Figure S7. Western blot results for Figure 7.
